# Biological Decline of Alfalfa Is Accompanied by Negative Succession of Rhizosphere Soil Microbial Communities

**DOI:** 10.3390/plants13182589

**Published:** 2024-09-16

**Authors:** Yuanyuan Ma, Yan Shen, Xiaoping Zhou, Hongbin Ma, Jian Lan, Bingzhe Fu, Quanhong Xue

**Affiliations:** 1 College of Forestry and Prataculture, Ningxia University, Yinchuan 750021, China; 13363921675@163.com (Y.M.); 18362098886@163.com (H.M.); 17395460813@163.com (J.L.); baizixi2022@163.com (B.F.); 2 Ningxia Rural Science and Technology Development Center, Yinchuan 750001, China; 15909546644@163.com; 3 College of Natural Resources and Environment, Northwest A&F University, Yangling 712100, China; xuequanhong@163.com

**Keywords:** *Medicago sativa* L., biological decline, rhizosphere-specific taxa, time-enriched taxa, time-depleted taxa, functional prediction

## Abstract

The growth and biological decline of alfalfa may be linked to the rhizosphere microbiome. However, plant–microbe interactions in the rhizosphere of alfalfa and associated microbial community variations with stand age remain elusive. This study explored the successional pattern of rhizosphere microbial communities across different aged alfalfa stands and its relationship with alfalfa decline. Rhizosphere soils were collected from 2- and 6-year-old alfalfa stands. Control soils were collected from interspaces between alfalfa plants in the same stands. Soil bacterial and fungal communities were characterized by 16S and ITS rRNA gene sequencing, respectively. Specific microbial taxa colonized the rhizosphere soils, but not the control soils. The rhizosphere-specific taxa mainly included potentially beneficial genera (e.g., *Dechloromonas*, *Verrucomicrobium*) in the young stand and harmful genera (e.g., *Peziza*, *Campylocarpon*) in the old stand. Alfalfa roots regulated soil microbial communities by selective promotion or inhibition of distinct taxa. The majority of time-enriched taxa were reported as harmful fungi, whose relative abundances were negatively correlated with plant traits. Time-depleted taxa were mostly known as beneficial bacteria, which had relative abundances positively correlated with plant traits. The relative abundances of functional bacterial genes associated with vancomycin biosynthesis, zeatin biosynthesis, and amino acid metabolism trended lower in rhizosphere soils from the old stand. An upward trend was observed for fungal pathogens and wood saprotrophs with increasing stand age. The results suggest that root activity drives the negative succession of rhizosphere microbial communities during alfalfa decline in old stands.

## 1. Introduction

Alfalfa (*Medicago sativa* L.) is a perennial leguminous forage crop widely grown in the United States, Europe, and Asia for hay and silage [1]. A long-term bottleneck in growing alfalfa is that biological decline occurs in old stands with increasing age. Alfalfa plants aged 2 years perform the best in yield and quality. When growing for more than four consecutive years, alfalfa plants become smaller and sparser, with reduced plant height, decreased branch number, lower root activity, and increased disease incidence. All these changes lead to a precipitous decline in the nutritional quality and yield of alfalfa [2,3]. Alfalfa decline is commonly seen across its growing areas in the loess hilly region of Northwest China, constraining the development of alfalfa production [4]. To reduce economic losses and achieve sustainable production, the alfalfa decline problem must be solved.

Many crops decline in terms of yield loss and quality reduction under long-term continuous cropping [5,6,7]. In most cases, crop decline is associated with abnormally structured rhizosphere microbiomes [7,8,9]. For example, long-term continuous cropping of flue-cured tobacco (*Nicotiana tabacum* L.) altered the composition of rhizosphere bacterial and fungal communities. With increasing years of continuous cropping, rhizosphere microbial taxa involved in soil elemental cycling (e.g., Chloroflexi, Patescibacteria) decreased in their relative abundances, which were correlated with the yield loss and quality reduction in tobacco [10]. Additionally, potentially harmful taxa (e.g., *Fusarium*) increased in the rhizosphere soil of American ginseng (*Panax quinquefolius* L.) under continuous cropping for three years, causing severe root rot [11]. Although previous studies have characterized rhizosphere microbial communities associated with alfalfa (e.g., [12]), whether they contain unique taxa that are distinct from those in bulk soil communities is still unclear. Does the structure of rhizosphere microbial communities vary with increasing stand age of alfalfa, and if yes, how do microbial community variations relate to alfalfa decline? Answering these questions can enhance our mechanistic understanding of alfalfa decline.

Rhizosphere microbes live at the plant root–soil interface. These taxonomically and functionally diverse microbes interact with host plants and play a crucial role in plant growth, development, and stress tolerance [13]. The rhizosphere microbiome is also referred to as the second genome of plants [14], and its structure is shaped by microbial interactions with roots and soil [15]. Specific host plants and soil types allow for the formation of rhizosphere-specific microbial communities [16]. On the one hand, beneficial microbes recruited to the rhizosphere can produce microbiostatic and microbicidal compounds, or activate the plant immune system to achieve an antimicrobial effect. On the other hand, pathogenic microbes recruited by root exudates are likely to compete for nutrients and impair plant growth, causing crop failure [17]. Therefore, characterizing the diversity and composition of rhizosphere microbial communities associated with alfalfa has implications for the long-term management of alfalfa growth, stress tolerance, and disease biocontrol.

In the present study, we hypothesized that alfalfa decline is accompanied by abnormal variations in the rhizosphere microbiome. The purpose of the study was to seek evidence that rhizosphere microbial communities are abnormally structured during alfalfa decline in old stands. Rhizosphere soils were collected from alfalfa stands of different ages, and microbial communities were characterized by 16S and ITS rRNA gene sequencing. The composition of rhizosphere-specific taxa was determined by comparing them with microbial taxa in control soils obtained from interspaces between alfalfa plants in the same stands. The successional pattern of rhizosphere microbial communities from young to old stand age was analyzed, and its association with alfalfa decline was explored. The results of this study could uncover the microbial mechanisms underlying alfalfa decline and provide useful data for the development of early detection and prevention techniques.

## 2. Results

### 2.1. Rhizosphere Microbial Community Diversity

**Figure 1:**
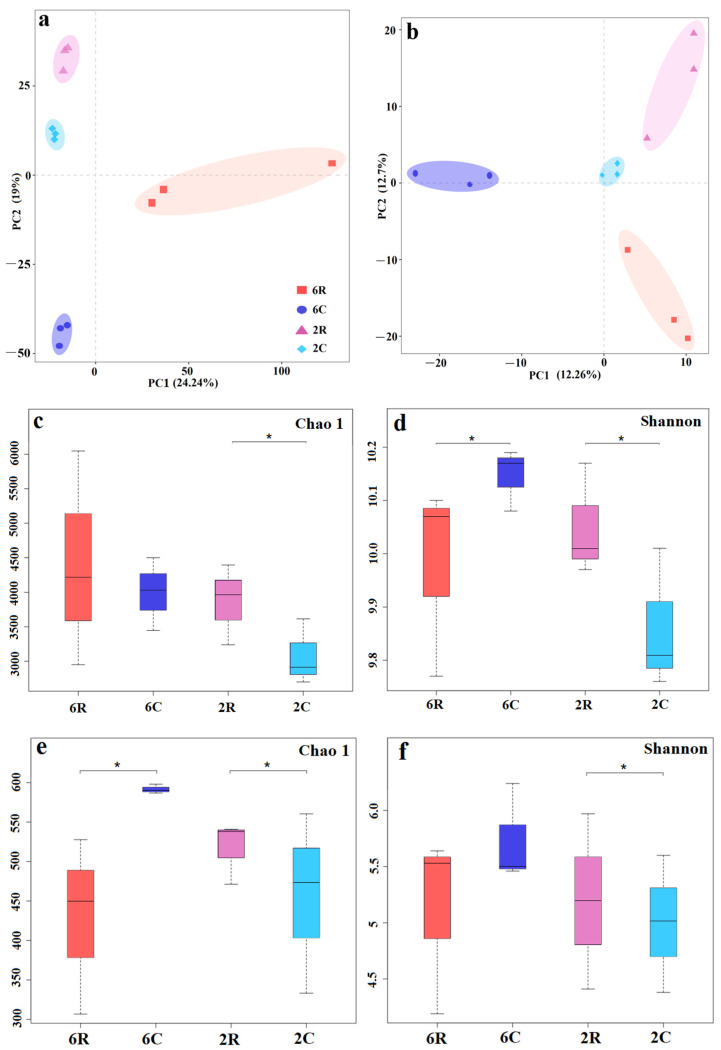
Soil microbial community diversity in alfalfa stands of different ages: (**a**,**b**) Principal coordinates analysis of bacterial and fungal β-diversity based on Bray–Curtis distance. (**c**,**d**) α-Diversity indices of bacteria. (**e**,**f**) α-Diversity indices of fungi. 2R and 2C are rhizosphere soils from the 2-year-old alfalfa stand and corresponding control soils, respectively; 6R and 6C are rhizosphere soils from the 6-year-old alfalfa stand and corresponding control soils, respectively. Error bars represent the standard deviation of the means (n = 3). * indicates a significant difference in α-diversity between rhizosphere and control soils (*p* < 0.05).

Sequence clustering yielded a total of 11,030 bacterial OTUs (40 phyla and 778 genera) and 1145 fungal OTUs (9 phyla and 232 genera). For both bacterial (Figure 1a) and fungal (Figure 1b) communities, PCA distinguished rhizosphere soil samples from the 6-year-old alfalfa stand (6R) and corresponding controls (6C), as well as rhizosphere soil samples from the 2-year-old alfalfa stand (2R) and corresponding controls (2C). Compared to 2C soils, 2R soils showed higher bacterial and fungal diversity in terms of Chao1 and Shannon indices (Figure 1c–f). Lower microbial diversity was observed for 6R soils compared to 6C soils (Figure 1d–f), except for the Chao1 index of bacteria (Figure 1c).

### 2.2. Identification of Rhizosphere-Specific Microbial Taxa

**Figure 2:**
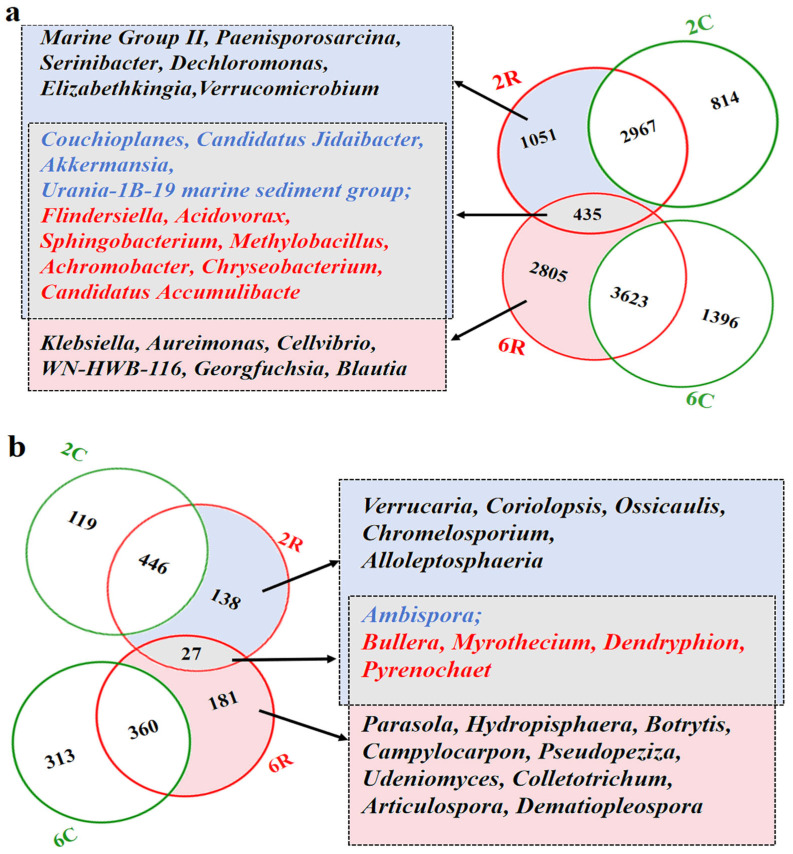
Microbial taxa enriched in the rhizosphere soil of alfalfa: (**a**) Number of operational taxonomic units (right) and taxonomy of genera (left) in the rhizosphere-specific bacterial community. (**b**) Number of operational taxonomic units (left) and taxonomy of genera (right) in the rhizosphere-specific fungal community. The blue color indicates genera with a higher relative abundance in rhizosphere soils from the 2-year-old alfalfa stand (2R) than in the corresponding control soils (2C), and the red color indicates genera with a higher relative abundance in rhizosphere soils from the 6-year-old alfalfa stand (6R) than in the corresponding control soils (6C).

Comparing the microbial community composition between sample groups (2R vs. 2C; 6R vs. 6C) revealed the presence of unique taxa in the rhizosphere soils, but not in the control soils (i.e., rhizosphere-specific taxa). In 2R soils, 1486 bacterial OTUs (Figure 2a) and 165 fungal OTUs (Figure 2b) were enriched, representing 98 bacterial and 18 fungal genera, respectively. In 6R soils, 3240 bacterial OTUs (Figure 2a) and 208 fungal OTUs (Figure 2b) were enriched, representing 124 bacterial and 39 fungal genera, respectively. Among the rhizosphere-specific taxa, 435 bacterial OTUs and 27 fungal OTUs were shared between 2R and 6R soils, which represented 11 bacterial and 5 fungal genera, respectively.

### 2.3. Regulation of Rhizosphere Microbial Communities by Root Activity

**Figure 3:**
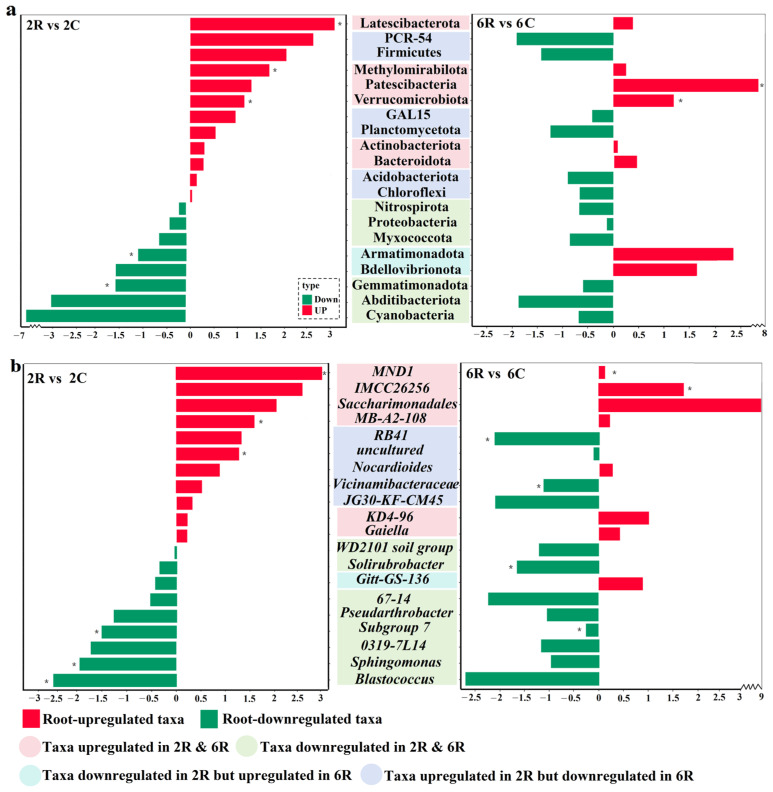
The selection of rhizosphere soil bacteria by root activity of alfalfa. Log_10_ fold changes in the relative abundance of the top 20 bacterial (**a**) phyla and (**b**) genera. 2R vs. 2C indicates the comparison group between rhizosphere soils from the 2-year-old alfalfa stand and corresponding control soils; 6R vs. 6C indicates the comparison group between rhizosphere soils from the 6-year-old alfalfa stand and corresponding control soils. * indicates a significant difference in taxa relative abundance between rhizosphere and control soils (*p* < 0.05).

To evaluate the selective effects of root activity on rhizosphere microbial communities, taxa with higher (i.e., root-upregulated taxa) or lower (i.e., root-downregulated taxa) relative abundances in rhizosphere soils than in control soils were analyzed. In both 2R and 6R soils, the bacterial community was dominated by the phylum Actinobacteriota, which accounted for 42.5% and 39.4%, respectively. The root effects on the top 20 most abundant bacterial phyla (Figure 3a) and genera (Figure 3b) were depicted. Irrespective of stand age, root activity upregulated the relative abundances of the phyla Latescibacterota and Methylomirabilota, as well as the genera *MND1* and *IMCC26256.* The opposite effect of root activity was observed for the phyla Cyanobacteria and Abditibacteriota, in addition to the genera *Blastococcus* and *Saccharomyces*. Interestingly, the phyla PCR-54 and Firmicutes, together with the genus *RB41*, were upregulated in 2R soils but downregulated in 6R soils. In contrast, the phyla Bdellovibrionota and Armatimonadota, as well as the genus *Gitt-GS-136*, were downregulated in 2R soils but upregulated in 6R soils.

**Figure 4:**
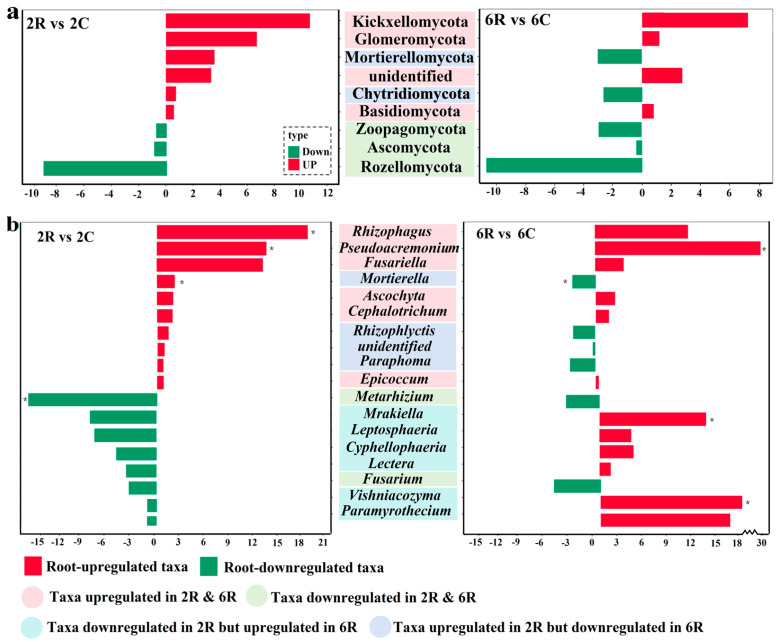
The selection of rhizosphere soil fungi by root activity of alfalfa. Log_10_ fold changes in the relative abundance of the nine fungal (**a**) phyla and (**b**) the top 19 most abundant genera. 2R vs. 2C indicates the comparison group between rhizosphere soils from the 2-year-old alfalfa stand and corresponding control soils; 6R vs. 6C indicates the comparison group between rhizosphere soils from the 6-year-old alfalfa stand and corresponding control soils. * indicates a significant difference in taxa relative abundance between rhizosphere and control soils (*p* < 0.05).

The fungal community in 2R and 6R soils was dominated by the phylum Ascomycota, which accounted for 58.6% and 69.6%, respectively. The root effects on the nine fungal phyla (Figure 4a) and the top 19 most abundant fungal genera (Figure 4b) were analyzed. In addition to the phyla Kickxellomycota and Glomeromycota, the genera *Rhizophagus* and *Fusariella* were upregulated by root activity, irrespective of stand age. The opposite effect of root activity was observed for the phyla Rozellomycota and Ascomycota (Figure 4a), as well as the genus *Metarhizium*. Some phyla (e.g., Mortierellomycota, Chytridiomycota) and genera (e.g., *Mortierella*, *Rhizophlyctis*) were upregulated in 2R soils, while they were downregulated in 6R soils. Most of the fungal genera downregulated in 2R soils were upregulated in 6R soils (e.g., *Paramyrothecium*, *Vishniacozyma*).

### 2.4. Effects of Stand Age on Rhizosphere Microbial Communities

**Figure 5:**
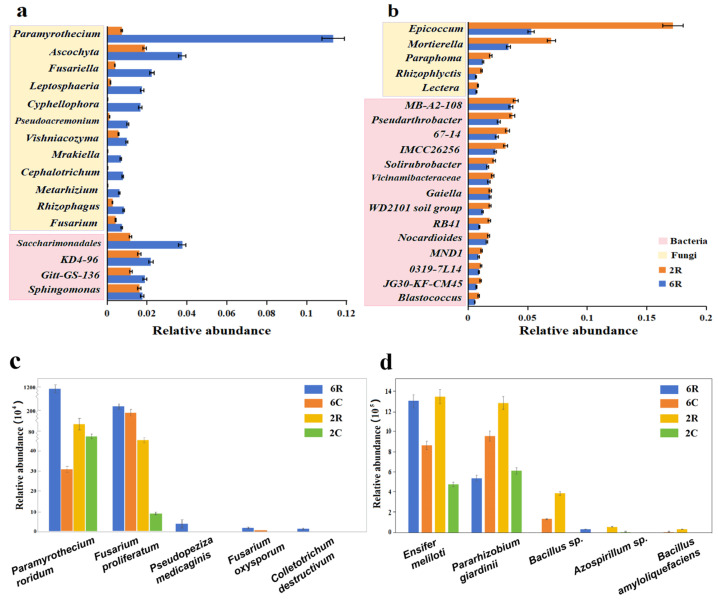
Relative abundances of time-enriched (**a**), time-depleted (**b**), pathogenic (**c**), and beneficial (**d**) microbial taxa in the rhizosphere soil of alfalfa. 2R and 2C represent rhizosphere soils from the 2-year-old alfalfa stand and corresponding control soils, respectively; 6R and 6C represent rhizosphere soils from the 6-year-old alfalfa stand and corresponding control soils, respectively. Error bars represent the standard deviation of the means (n = 3).

The response of rhizosphere microbial communities to stand age was characterized by focusing on taxa with higher (i.e., time-enriched taxa) or lower (i.e., time-depleted taxa) relative abundance in 6R soils than in 2R soils. Among the top 20 bacterial and fungal genera in terms of relative abundance, the time-enriched taxa were mainly fungi (Figure 5a), whereas the time-depleted taxa were mostly bacteria (Figure 5b). At the species level, pathogenic microbes, such as *Paramyrothecium roridum*, *Fusarium oxysporum*, and *Pseudopeziza medicaginis*, emerged as time-enriched taxa (Figure 5c). Beneficial microbes, represented by *Ensifer meliloti*, *Pararhizobium giardinii*, and *Bacillus* spp. were identified as time-depleted taxa (Figure 5d).

### 2.5. Predicted Functions of Rhizosphere Microbial Communities

**Figure 6:**
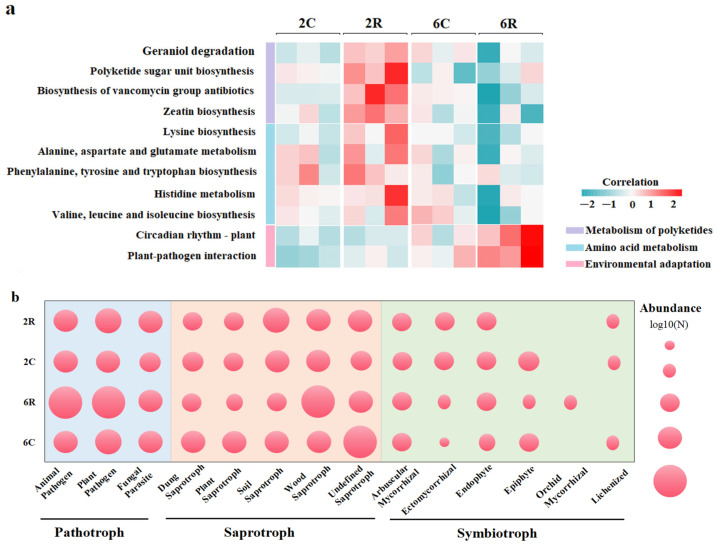
Functional shifts of rhizosphere bacterial (**a**) and fungal (**b**) communities associated with alfalfa. 2R and 2C denote rhizosphere soils from the 2-year-old alfalfa stand and corresponding control soils, respectively; 6R and 6C represent rhizosphere soils from the 6-year-old alfalfa stand and corresponding control soils, respectively.

Picrust2 predicted 176 functional categories of bacterial communities in all soil samples. Some bacterial functional genes related to important metabolic pathways showed significantly different relative abundances among various samples (Figure 6a). In 6R soils, bacterial functional genes related to environmental adaptation increased compared to those of other soils, whereas genes involved in polyketide metabolism and amino acid metabolism decreased in 6R soils. FunGuild-based functional prediction of fungi yielded three trophic modes (pathotroph, saprotroph, and symbiotroph). Using the Kruskal–Wallis rank sum test, 14 fungal guilds were detected at different relative abundances among samples (Figure 6b). Compared to 6C and 2R soils, pathogens and wood saprotrophs increased in 6R soils, accompanied by decreased plant saprotrophs, soil saprotrophs, and epiphytes. Ectomycorrhizal fungi also increased in 6R soils compared to 2R soils, whereas lichenized fungi almost disappeared in 6R soils.

### 2.6. Linking Rhizosphere Microbial Taxa to Plant Traits and Soil Properties

**Figure 7:**
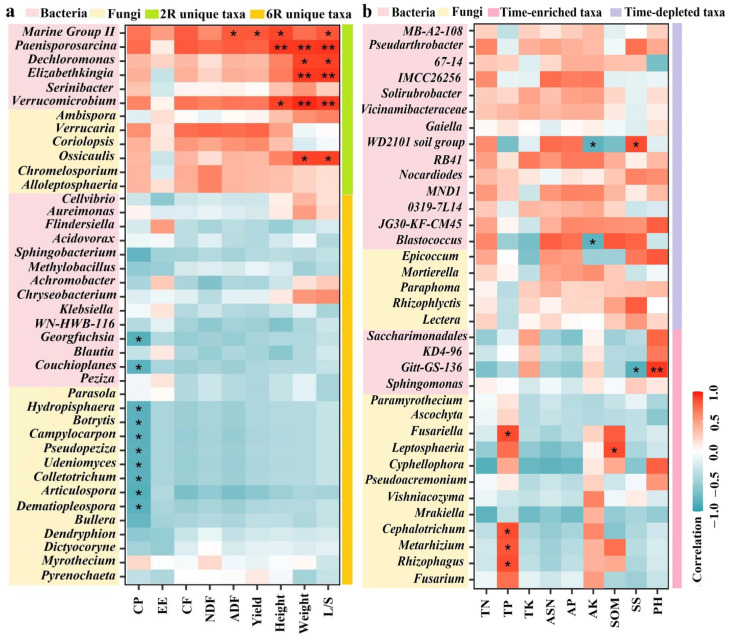
Correlation heatmaps between rhizosphere microbial taxa and plant traits of alfalfa: (**a**) Rhizosphere-specific taxa; (**b**) Time-dependent taxa. 2R and 6R denote rhizosphere soils from the 2- and 6-year-old alfalfa stands, respectively. CP, crude protein; EE, ester extract; CF, crude fiber; NDF, neutral detergent fiber; ADF, acid detergent fiber; L/S, leaf-to-stem ratio. * *p* < 0.05; ** *p* < 0.01.

To clarify the relationship of rhizosphere-specific taxa, time-enriched taxa, and time-depleted taxa to plant traits and soil properties, Spearman’s correlation heatmaps were created (Figure 7 and Figure S1). At the genus level, taxa unique to 2R soils (Figure 7a and Figure S1a) and time-depleted taxa (Figure 7b and Figure S1b) mostly showed positive correlations with plant traits and soil properties. For example, *Paenisporosarcina* and *Verrucomicrobium* (rhizosphere-specific bacteria) were significantly positively correlated with plant height, fresh weight, and leaf-to-stem ratio (*p* < 0.05). *Paenisporosarcina* was also significantly positively correlated with soil alkali-hydrolyzable nitrogen and available phosphorus contents (*p* < 0.05). Additionally, a significant positive correlation emerged between *Blastococcus* (time-depleted bacteria) and plant height (*p* < 0.05). *Epicoccum* and *Rhizophlyctis* (time-depleted fungi) were significantly positively correlated with plant fresh weight and leaf-to-stem ratio, respectively (*p* < 0.05). Generally, taxa unique to 6R soils (Figure 7a and Figure S1a) and time-enriched taxa (Figure 7b and Figure S1b) showed negative correlations with plant traits and soil properties. For example, *Botrytis*, *Campylocarpon*, and *Colletotrichum* (rhizosphere-specific fungi) were significantly negatively correlated with crude protein content in shoots (*p* < 0.05). *Campylocarpon* and *Alloleptosphaeria* (rhizosphere-specific fungi) were significantly negatively correlated with soil total potassium content. *Cyphellophora* (time-enriched fungi) was significantly negatively correlated with plant height, as well as crude protein and neutral detergent fiber contents in shoots (*p* < 0.05).

## 3. Discussion

This study indicated that alfalfa root activity exhibited selective promoting or inhibitory effects on rhizosphere microbial communities. Consequently, the rhizosphere soil of alfalfa was colonized by unique microbial taxa, and their composition varied with stand age. While some rhizosphere taxa were enriched in the old stand compared to the young stand, others were depleted. The relative abundance and function of time-enriched and time-depleted taxa were closely linked to plant growth, yield, and quality traits of alfalfa. These rhizosphere microbial taxa may serve as potential biomarkers for early detection or even prediction of biological decline in alfalfa. The results also provide useful data to select potential functional microbes for the prevention of alfalfa decline by manipulation of the rhizosphere microbiome.

### 3.1. Variations in Rhizosphere-Specific Microbial Communities with Stand Age

In the present study, specific microbial taxa were found to colonize the rhizosphere of alfalfa, depending on stand age. In the young stand, the rhizosphere-specific taxa were represented by the genera *Marine Group II*, *Dechloromonas*, and *Verrucomicrobium* (bacteria), as well as *Verrucaria* and *Coriolopsis* (fungi). These taxa showed beneficial effects on plant growth, yield, and quality traits, and some of them were closely related to soil nutrient availability. *Dechloromonas* is a group of denitrifying bacteria, which can also inhibit plant pathogens [18]. *Verrucomicrobium* has been reported to facilitate the establishment of beneficial plant–microbe interactions by maize roots in a rainfed agroecosystem [19]. In the old stand, the rhizosphere-specific taxa were mainly identified as *Peziza* (bacteria), *Campylocarpon*, *Pseudopeziza*, *Colletotrichum*, and *Botrytis* (fungi). Most of these taxa exhibited adverse effects on alfalfa quality in terms of crude protein content. Notably, the four fungal taxa comprise pathogens commonly found in crops [20,21,22,23]. *P. medicaginis* of the genus *Pseudopeziza* is the principal pathogen causing common leaf spot in alfalfa [24].

To sum up, the rhizosphere-specific taxa detected in the young alfalfa stand played a positive role in enhancing plant growth. In the old alfalfa stand, more harmful taxa were unique to the rhizosphere soil. This community shift driven by stand age could be attributed to the selective effects of alfalfa root activity.

### 3.2. Selective Promotion and Inhibition of Rhizosphere Microbial Taxa by Root Activity

Root activity promoted a set of bacteria to colonize the rhizosphere of alfalfa across stand ages, as demonstrated by their upregulated relative abundances. The root-upregulated bacteria mainly included the phyla Latescibacterota, Methylomirabilota, Patescibacteria, Verrucomicrobiota, and Actinobacteriota, as well as the genera *MND1*, *Saccharimonadales*, and *KD4-96*. Methylomirabilota methanotrophs play a role in plant nitrogen fixation [25]. Patescibacteria species can enhance cadmium tolerance in *Dahlia pinnata* Cav. [26]. The phylum Actinobacteriota has been reported to facilitate plant growth and reduce the harm of environmental stresses [27]. *MND1* is a genus associated with suppression of cucumber wilt [28]. The genus *Saccharimonadales* is a major contributor to the availability of soil phosphorus [29]. *KD4-96* is a genus of aerobic beneficial fungi [30]. Root activity also promoted rhizosphere colonization by some fungi, including the phyla Kickxellomycota, Glomeromycota, and Basidiomycota, as well as the genera *Rhizophagus*, *Pseudoacremonium*, and *Fusariella*. Glomeromycota is a phylum of arbuscular mycorrhizal fungi, providing essential nutrient support for plant growth [31]. The genus *Rhizophagus* can establish mutualistic symbiosis with legume roots to form root nodules and fix nitrogen. *Rhizophagus* spp. are also able to alleviate leaf spot and bolster plant resistance to pea aphids in alfalfa [32]. The genus *Fusariella* contains a variety of plant pathogens [33], and several species of the genus *Ascochyta* are known as plant pathogens [34].

Some bacteria were found to be inhibited by alfalfa root activity across stand ages, as indicated by their downregulated relative abundances. The root-downregulated bacteria were represented by the phyla Cyanobacteria and Myxococcota, and the genera *Blastococcus*, *Sphingomonas*, and *Pseudarthrobacter*. The phylum Myxococcota plays a prominent role in the rhizosphere biological process of rice allelopathy, and its proportion is higher in the rhizosphere of rice plants with stronger allelopathy [35]. The genus *Blastococcus* shows higher relative abundance in maize rhizosphere under continuous cropping than under rotational cropping [36]. *Sphingomonas* spp. perform beneficial functions, such as the degradation of complex organic matter and antagonism of plant pathogenic fungi [37]. *Pseudarthrobacter* spp. show plant growth-promoting effects [38]. The fungi inhibited by alfalfa root activity mainly belonged to the phyla Rozellomycota and Ascomycota, in addition to the genus *Metarhizium*. Most known plant pathogens belong to the phylum Ascomycota [39,40], whereas *Metarhizium* spp. can colonize roots to promote plant growth [41].

In summary, alfalfa root activity regulated rhizosphere microbial communities by selectively promoting or inhibiting the colonization of distinct taxa. Most of the root-upregulated taxa conveyed beneficial effects on host plants, although there were also some pathogens. While the root-downregulated taxa mainly exhibited harmful effects on host plants, some were known as beneficial taxa. The selection of soil microbes by root activity could be related to the stand age and growth environment of alfalfa.

### 3.3. Rhizosphere Microbial Community Succession in Relation to Alfalfa Decline

As the stand age of alfalfa increased, the rhizosphere-specific microbial communities changed to be dominated by potentially harmful taxa, and the root effects on microbial taxa varied. For the bacterial community, the phyla Firmicutes and Planctomycetota, and the genera *Nocardiodes*, *RB41*, and *JG30-KF-CM45* were promoted in the young stand but inhibited in the old stand. The same pattern was observed for the phylum Chytridiomycota and the genera *Mortierella* and *Rhizophlyctis* in the fungal community. It has been reported that *Nocardiodes* can enhance the availability of soil iron and potassium, improving plant nutrition [42]. *JG30-KF-CM45* is a group of denitrifying bacteria [43]. *Mortierella* can facilitate crop growth by increasing the contents of soil available phosphorus, potassium, calcium, magnesium, and boron [44]. The majority of taxa inhibited in the young stand were promoted in the old stand, including the phyla Bdellovibrionota and Armatimonadota and the genus *Gitt-GS-136* (bacteria), as well as the genera *Paramyrothecium* and *Vishniacozyma*, *Leptosphaeria*, *Cyphellophaeria*, and *Mrakiella* (fungi). *Paramyrothecium* spp. are reported as plant pathogens and cause leaf spot or leaf blight on many commercial crops worldwide [45]. *Leptosphaeria* spp. cause phoma leaf spot and stem cancer in many plant species [46,47]. This means that old alfalfa plants created favorable conditions for rhizosphere colonization by harmful microbes, rather than beneficial microbes.

As time-enriched species, a range of pathogenic bacteria (e.g., *P. medicaginis*, *C. destructivum*, *P. roridum*, and *Fusarium* spp.) was negatively related to plant traits. *C. destructivum* and *F. oxysporum* are important pathogens responsible for root rot of alfalfa [48]; *P. roridum* is the primary pathogen causing common leaf spot of alfalfa [49]. As time-depleted species, some beneficial bacteria (e.g., *E. meliloti*, *P. giardinii*, and *Bacillus amyloliquefaciens*) were positively related to plant traits. *E. meliloti* and *P. giardinii* are major nitrogen-fixing rhizobia associated with alfalfa [50]. Both of the species can promote plant growth and mediate rhizosphere communities [51]. Additionally, *B. amyloliquefaciens* is a crucial biocontrol bacterium, which has the ability to increase crop yield, inhibit pathogen growth, and reduce plant diseases [52].

### 3.4. Rhizosphere Microbial Community Function Associated with Alfalfa Decline

An increase in bacteria related to environmental adaption was observed in rhizosphere soils from the old stand. This indicates deterioration of the rhizosphere microenvironment and consequent enrichment of bacteria with stronger adaptive capacity. Amino acid-metabolizing bacteria also decreased in rhizosphere soils from the old stand. Amino acids are raw materials for protein biosynthesis; these compounds play a decisive role in plant growth by enhancing physiological and biochemical processes, such as photosynthesis, chlorophyll biosynthesis, nutrient uptake, and root development [53]. As amino acid metabolism is involved in plant nitrogen cycling [54], the decrease in amino acid-metabolizing bacteria is indicative of a potential decline in nitrogen cycling function in the rhizosphere microenvironment of old alfalfa plants.

Plant–pathogen interactions are regulated by the genotype and biochemical traits of both the plant and the pathogen [55]. Polyketide-metabolizing bacteria decreased in rhizosphere soils from the old stand, as evidenced by the lower relative abundances of taxa related to vancomycin biosynthesis, zeatin biosynthesis, and geranylgeraniol degradation. This provides evidence that the old alfalfa stand is less resistant to pathogen stress and more susceptible to pathogen attack. Polyketides are a class of natural secondary metabolites with biological activity produced by microbes and plants. These compounds are of biological significance in plant–environment interactions, as well as in plant growth and development [56]. In particular, vancomycin is a glycopeptide antibiotic that prevents the biosynthesis of bacterial cell walls and shows bactericidal effects [8]. As a cell growth regulator, zeatin performs functions in promoting lateral bud growth, stimulating cell differentiation, accelerating callus and seed germination, and preventing leaf senescence [57].

Fungal pathogens, wood saprotrophs, and orchid mycorrhizal fungi preferentially colonized rhizosphere soils from the old stand. Fungal pathogens impair host root cells to acquire nutrients, causing soil-borne diseases; a higher diversity of fungal pathogens leads to a lower stability of ecosystem productivity [58]. Wood saprotrophs thrive in plant litter, degrading lignin and cellulose [59]. The higher abundance of wood saprotrophs in the old alfalfa stand may be attributed to the accumulation of fallen stems and leaves, together with aged and dead roots in the rhizosphere soil. Plant saprotrophs, soil saprotrophs, and ectomycorrhizal fungi were inhibited to some extent in rhizosphere soils from the old stand. Saprophytic fungi decompose and utilize organic matter, acting as one of the key drivers of soil nutrient transformation [58]. Ectomycorrhizal fungi help plants to absorb nutrients and resist pathogen attack [60]. Therefore, the possible reasons for alfalfa decline include the increase in pathogenic microbes and the decrease in beneficial microbes in the rhizosphere soil, coupled with the weakening of important metabolic functions.

## 4. Materials and Methods

### 4.1. Study Area

The study was conducted in Pengyang County (106°38′ E, 35°4l′ N), a rainfed agricultural area in the loess hilly region of southern Ningxia, China. This area is located in the central hilly gully area of the Loess Plateau, with a typical temperate semi-arid continental monsoon climate. The mean annual temperature ranges from 7.4 °C to 8.5 °C, and the mean annual precipitation is 429.8 mm. The major soil type is classified as loessial soil (Entisol). Since the national policy of returning farmland to forest and grassland was implemented in 2003, alfalfa has been widely grown in this area. The experiment began in April 2017 and lasted until May 2023. We selected two field plots (48 m × 12 m each) with the same elevation and spaced ~100 m apart in the study area. One plot was grown with alfalfa in 2017, after 2 years of maize cropping; this plot was referred to as the 6-year-old alfalfa stand (old stand). The other plot was grown with alfalfa in 2021, after 6 years of maize cropping; this plot was referred to as the 2-year-old alfalfa stand (young stand). The alfalfa cultivar used in the experiment was Gannong No. 4. The plots were managed under the same cropping system and practices, including annual harvest time, fertilization, and irrigation. Sampling was carried out at the first full-bloom stage of alfalfa in May 2023. Tables S1 and S2 provide details for the alfalfa plant traits and soil properties in the experimental plots.

### 4.2. Soil Sampling and Chemical Analysis

Three 1 m × 1 m quadrats were randomly selected from each stand. In each quadrat, soil profiles were dug to 30 cm depth using a shovel, within 50 cm from the stem of three randomly selected alfalfa plants. Alfalfa roots were exposed as completely as possible, and the loose soil adhering to fibrous roots was removed with a soil knife. Then, the fibrous roots were gently shaken and the fallen soil was collected (50 g per plant). Soils from the roots of three plants were thoroughly mixed and used for chemical analysis. The remaining soil adhering to roots (i.e., rhizosphere soil) was brushed off with a sterilized hairbrush and passed through a 20-mesh sterilized sieve. After removing impurities with sterilized forceps, rhizosphere soils from three plants were thoroughly mixed, then sealed in sterile centrifuge tubes (15.00 g per tube) and immediately stored at −80 °C until DNA extraction. To collect control soils, three patchy areas with no weeds and no alfalfa plants were selected in each stand. After removing ~1 cm thick topsoil, soils were collected from the 0–30 cm depth with a 7.5 cm diameter soil auger. The control soils were prepared as described for the rhizosphere soils.

Soil samples were air-dried, ground, and passed through a 20-mesh sieve before chemical analysis. The potassium dichromate volumetric method was used to determine soil organic matter [61]. For quantification of alkali-hydrolyzable nitrogen and total nitrogen, the alkaline hydrolysis and semi-micro Kjeldahl methods were respectively used [62]. Soil total phosphorus and potassium were respectively analyzed by molybdenum-antimony anti-colorimetric assay and flame photometry with samples fused using sodium hydroxide. Ultraviolet spectrophotometry was conducted to analyze soil available phosphorus in sodium bicarbonate extracts [63]. Flame photometry was adopted to determine soil-available potassium in ammonium acetate extracts [64]. Soil pH was measured in 1:2 (*w*/*v*) soil suspensions using a digital pH meter (PHST-5; Merweather Biotechnology Co., Ltd., Shanghai, China). Soil total salt was determined by conductometry [65].

### 4.3. Soil Microbial Community Analysis

Genomic DNA extraction from soil samples (5.00 g each) was performed using the E.Z.N.A. Soil DNA Kit (Omega Bio-tek, Inc., Doraville, GA, USA). After quality check, the extracted DNA served as a template for amplifying the V3–V4 region of the bacterial 16S rRNA gene and the internal transcribed spacer 1 (ITS1) region of the fungal 18S nuclear RNA gene. The PCR primers used were 338F (5’-ACTCCTACGGGGAGGCAGCAG-3’) and 806R (5’-GGACTACHVGGGTWTCTAAT-3’) for bacteria [66], and SSU0817F (5’-TTAGCATGGAATAATRRAATAGGA-3’) and 1196R (5’-TCTGGACCTGGTGAGTTTCC-3’) for fungi [67]. PCR products were purified automatically using the Agencourt AMPure XP Kit (Beckman Coulter, Inc., Brea, CA, USA). Library construction was performed using the NEB Next Ultra II DNA Library Prep Kit (New England Biolabs, Inc., Ipswich, MA, USA). The libraries were sequenced on Illumina Novaseq 6000 (Illumina, Inc., San Diego, CA, USA) to generate 2 × 250 bp paired-end reads. The raw data were obtained in FASTQ format with truncated adapter sequences and have been deposited in the NCBI Sequence Read Archive database (https://www.ncbi.nlm.nih.gov/; accessed on 17 June 2024) under project numbers PRJNA1124538 (bacteria) and PRJNA1124542 (fungi).

### 4.4. Plant Sampling and Analysis

In each quadrat, six alfalfa plants were randomly selected and completely dug out for growth analysis. The shoots of alfalfa plants remaining in the six quadrats were harvested and used for yield and quality evaluation. All plant samples were sealed in plastic bags and brought back to the laboratory. The fresh weight per plant and the total fresh weight of all plants from six quadrats were determined using an electronic scale (accuracy: 0.01 g). Alfalfa yield was calculated by dividing the total fresh weight of all plants by the quadrat area. Plant height was measured using a ruler, and stem thickness was obtained using digital Vernier calipers. The stems and leaves were separated, deactivated at 105 °C for 1 h, and oven-dried at 70 °C for 48 h until constant weight. The dry weight of stems and leaves was determined to calculate the leaf-to-stem ratio. After deactivation (105 °C, 24 h) of shoot samples, crude protein content was analyzed according to AOAC standards [68]. Crude fiber content was analyzed using the filter bag technique [69]. Neutral and acid detergent fiber contents were measured using an ANKOM A200i fiber analyzer (ANKOM Technology, Macedon, NY, USA) based on the method of Soest et al. [70]. Crude fat (ether extract) was determined using an ANKOM AXT15i automatic fat analyzer (ANKOM Technology) as described by Li et al. [71].

### 4.5. Data Analysis

Trimmomatic was used to denoise and assemble the raw reads; then, FLASH was used to perform precise denoising of the assembled reads [72]. Sequence analysis was carried out using QIIME v1.9.1 [73], and high-quality reads were obtained for downstream analysis. The high-quality reads were clustered into operational taxonomic units (OTUs) at 97% similarity using Uparse software (Uparse v7.0.1001) [74]. A representative sequence of OTUs was selected based on the principle of its algorithm, the sequence with the highest frequency of occurrence in OTUs was selected as the representative sequence of OTUs. Species annotation of OTUs sequences was analyzed using the Mothur method with the SSUrRNA database from SILVA1384 (setting a threshold of 0.8 to 1) [75] to obtain taxonomic information and at each taxonomic level separately: kingdom, phylum, class, order, family, genus, species, and the community composition of each sample. Rapid multiple sequence alignment was performed using MUSCLE (Version 3.8.31) software to obtain phylogenetic relationships for all OTUs representative of the sequences. Based on OTU abundance data, the α- and β-diversity of bacterial and fungal communities were calculated and plotted using QIIME v1.9.1 [73]. Wilcoxon rank sum test of α-diversity indices and principal coordinates analysis (PCoA) of β-diversity were created using the ggplot2 package of R v3.6.0, and Venn diagrams of microbial communities were plotted using the Venn package of R v3.6.0 [76]. Bar charts of taxa relative abundance (Log_10_ fold changes) were plotted using Sigmaplot v15.0 (Systat Software Inc., San Jose, CA, USA). PICRUSt2 was adopted to predict bacterial community functions [77], and the FUNGuild database was used for prediction of fungal community functions [78]. Significant differences in the relative abundances of predicted functional pathways among samples were analyzed by the Kruskal–Wallis rank sum test [79]. The relationships of rhizosphere microbial taxa-to-plant traits and soil properties were determined using Spearman’s correlation analysis.

## 5. Conclusions

This study clarified the pattern of microbial community succession in the rhizosphere soil of alfalfa with stand age. The relationship between rhizosphere-specific microbial taxa and alfalfa biological decline was deciphered. It was found that root activity altered the composition of rhizosphere microbial communities by inhibiting or promoting colonization of distinct taxa. The selective effect of alfalfa roots varied with stand age. Overall, long-term continuous cropping of alfalfa led to a negative succession of rhizosphere microbial communities. This was evidenced by the enrichment of harmful fungi and the depletion of beneficial bacteria in the rhizosphere soil, coupled with the weakening of amino acid metabolism and antibiotic biosynthesis. The variations in the rhizosphere microbial community structure and function were linked to alfalfa decline in the old stand. The results of this study underscore the role of plant–microbe interactions in alfalfa decline. The findings could be useful for the development of biotechniques to detect and prevent alfalfa decline.

## Data Availability

All data generated or analyzed during this study are included in this published article [and its Supplementary Information files].

## Supplementary Materials

The following supporting information can be downloaded at: https://www.mdpi.com/article/10.3390/plants13182589/s1, Figure S1: Correlation heatmaps between rhizosphere microbial taxa and soil properties in alfalfa stands; Table S1: Plant traits of alfalfa; Table S2: Soil properties; Table S3: Relative abundance of unique microbial taxa in the rhizosphere soil of alfalfa (×10^6^); Table S4: Relative abundance of bacterial phyla in 2R and 2C (×10^4^); Table S5: Relative abundance of bacterial phyla in 6R and 6C (×10^4^); Table S6: Relative abundance of bacterial genera in 2R and 2C (×10^4^); Table S7: Relative abundance of bacterial genera in 6R and 6C (×10^4^); Table S8: Relative abundance of fungal phyla in 2R and 2C (×10 ^4^); Table S9: Relative abundance of fungal phyla in 6R and 6C (×10 ^4^); Table S10: Relative abundance of fungal genera in 2R and 2C (×10^5^); Table S11: Relative abundance of fungal genera in 6R and 6C (×10^5^); Table S12: Relative abundances of pathogenic microbial taxa in the rhizosphere soil of alfalfa (×10^6^); Table S13: Relative abundances of beneficial microbial taxa in the rhizosphere soil of alfalfa (×10^6^).
