# Supplementary material for: Biological Decline of Alfalfa Is Accompanied by Negative Succession of Rhizosphere Soil Microbial Communities
Source: Plants (Basel). 2024 Sep 16;13(18):2589. doi: 10.3390/plants13182589 (PMC11434760; doi:10.3390/plants13182589)
Supplement: Supplementary file 1 [file plants-13-02589-s001.zip › plants-3167415-supplementary.pdf]

Table S1. alfalfa traits and yield

	**CP (%)**	**EE (%)**	**CF (%)**	**NDF (%)**	**ADF (%)**	**Height (cm)**	**Weight (g/plant)**	**L/S (%)**	**Yield （kg/m²）**
**Young stand**	19.64±2.13a	1.38±0.52a	28.91±3.24a	34.69±4.67a	29.67±3.48a	71.5±10a	14.0±6.3a	3.4±1.2	1.85±0.54a
**Old stand**	17.02±1.20b	1.43±0.33a	26.02±2.95b	33.85±5.02a	28.26±0.15a	64.1±12b	7.2±5.4b	2.5±0.6	1.08±0.60b
**Δ%**	-13.3	3.6	-10.0	-2.4	-4.8	-10.3	-48.6	-26.5	-41.6

Different lowercase letters in the same column indicate significant difference ($P < 0.05$). (the same below)

Table S2. Soil properties

	TN (g/kg)	TP (g/kg)	TK (g/kg)	AN (mg/kg)	AK (mg/kg)	AP (mg/kg)	TOC (g/kg)	SS (g/kg)	pH
**2C**	0.68±0.22b	0.61±0.12a	13.54±1.35a	27.41±0.10a	56.42±1.25b	9.79±2.88b	11.44±3.45ab	0.2±0.05a	8.43±0.11a
**2R**	0.73±0.41a	0.64±0.08a	13.76±2.03a	31.4±2.56a	56.21±3.66b	13.84±0.69a	10.67±2.36b	0.25±0.08	8.28±0.05a
**6C**	0.87±0.19a	0.67±0.10a	14.6±0.95a	36.97±1.02a	85.12±2.39a	7.75±2.75b	13.87±0.29a	0.22±0.01	8.39±0.08a
**6R**	0.66±0.31b	0.64±0.25a	13.78±1.48a	24.47±3.05b	75.17±2.16a	6.71±1.45b	10.68±2.88b	0.22±0.04	8.35±0.15a

TN, Total nitrogen; TP, Total phosphorus; TK, total potassium; AN, available nitrogen; AK, available potassium; AP, available phosphorus; TOC, Total organic content; SS, Soil salt.

Table S3. Relative abundance of unique microbial taxa in the rhizosphere soil of alfalfa（×10^6）

	Bacterial genera	6R	6C	2R	2C
1	*Peziza*	14740	0	0	0
2	*Klebsiella*	197	0	0	0
3	*Aureimonas*	178	0	0	0
4	*Cellvibrio*	172	0	0	0
5	*WN-HWB-116*	153	0	0	0
6	*Georgfuchsia*	146	0	0	0
7	*Blautia*	114	0	0	0
8	*Flindersiella*	254	0	15	0
9	*Acidovorax*	223	0	31	0
10	*Sphingobacterium*	846	0	161	0
11	*Methylobacillus*	70	0	23	0
12	*Achromobacter*	89	0	38	0
13	*Chryseobacterium*	407	0	367	0
14	*Marine Group II*	0	0	283	0
15	*Paenisporosarcina*	0	0	161	0
16	*Serinibacter*	0	0	138	0
17	*Dechloromonas*	0	0	115	0
18	*Elizabethkingia*	0	0	115	0
19	*Verrucomicrobium*	0	0	107	0
20	*Couchioplanes*	13	0	15	0

Table S4. Relative abundance of bacterial phyla in 2R and 2C（×10^4）

Upregulated					Downregulated				
	bacterial phyla	2R	2C	ΔC（%）		bacterial phyla	2R	2C	ΔC（%）
1	Latescibacterota	22	11	100.0*	1	Cyanobacteria	26	129	-79.5*
2	RCP2-54	18	10	80.0	2	Abditibacteriota	6	12	-46.2
3	Firmicutes	62	39	59.0	3	Gemmatimonadota	328	466	-29.5*
4	Methylomirabilota	58	40	45.6*	4	Bdellovibrionota	32	45	-29.4
5	Patescibacteria	145	108	34.1	5	Armatimonadota	22	28	-19.1
6	Verrucomicrobiota	254	193	31.4*	6	Myxococcota	184	208	-11.8
7	GAL15	22	18	20.6	7	Proteobacteria	1723	1846	-6.6
8	Planctomycetota	369	329	12.2	8	Nitrospirota	30	31	-2.7
9	Actinobacteriota	4266	4096	4.1					
10	Bacteroidota	449	432	3.9					
11	Acidobacteriota	1000	978	2.2					
12	Chloroflexi	939	932	0.7					

* indicates significant difference in taxa relative abundance between rhizosphere and control soils ($P < 0.05$). (the same below)

Table S5. Relative abundance of bacterial phyla in 6R and 6C（×10^4）

Upregulated					downregulated				
	**bacterial phyla**	**6R**	**6C**	**ΔC（%）**		**bacterial phyla**	**6R**	**6C**	**ΔC（%）**
1	*Patescibacteria*	416	71	486.1*	1	*RCP2-54*	11	17	-35.5
2	*Armatimonadota*	14	8	71.9	2	*Abditibacteriota*	6	9	-33.3
3	*Bdellovibrionota*	38	26	46.3	3	*Firmicutes*	48	67	-27.6
4	*Verrucomicrobiota*	263	199	32.1*	4	*Planctomycetota*	241	322	-25.1
5	*Bacteroidota*	575	517	11.4	5	*Acidobacteriota*	804	978	-17.9
6	*Methylomirabilota*	42	40	5.0	6	*Myxococcota*	193	227	-14.7
7	*Latescibacterota*	16	15	4.9	7	*Cyanobacteria*	132	153	-13.6
8	*Proteobacteria*	1963	1939	1.2	8	*Chloroflexi*	882	1012	-12.8
9	*Actinobacteriota*	3943	3921	0.6	9	*Nitrospirota*	25	29	-12.1
					10	*Gemmatimonadota*	332	376	-11.7
					11	*GAL15*	18	20	-10.1

Table S6. Relative abundance of bacterial genera in 2R and 2C（×**10**^{4}）

upregulated					downregulated				
	**bacterial genera**	**2R**	**2C**	**ΔC（%）**		**bacterial genera**	**2R**	**2C**	**ΔC（%）**
1	*MND1*	22	11	90.2*	1	*Blastococcus*	89	159	-44.0*
2	*IMCC26256*	18	10	58.0	2	*Sphingomonas*	163	253	-35.7*
3	*Saccharimonadales*	62	39	44.9	3	*0319-7L14*	108	159	-32.0
4	*MB-A2-108*	58	40	31.0*	4	*Subgroup_7*	84	119	-29.1*
5	*RB41*	145	108	20.3	5	*Pseudarthrobacter*	380	505	-24.8
6	*uncultured*	254	193	15.3*	6	*67-14*	320	363	-11.8
7	*Nocardioides*	22	18	9.2	7	*Gitt-GS-136*	119	130	-8.3
8	*Vicinamibacteraceae*	369	329	8.9	8	*Solirubrobacter*	218	234	-7.1
9	*JG30-KF-CM45*	105	98	7.0	9	*WD2101_soil_group*	187	189	-1.2
10	*KD4-96*	161	153	5.0					
11	*Gaiella*	192	184	4.1					

Table S7. Relative abundance of bacterial genera in 6R and 6C（×10^4）

Upregulated					downregulated				
	**bacterial genera**	**6R**	**6C**	**ΔC（%）**		**bacterial genera**	**6R**	**6C**	**ΔC（%）**
1	*Saccharimonadales*	377	47	704.2*	1	*Blastococcus*	51	96	-46.9*
2	*IMCC26256*	230	156	47.2*	2	*67−14*	240	402	-40.3
3	*KD4-96*	220	175	25.5	3	*RB41*	94	152	-38.0
4	*Gitt-GS-136*	189	154	22.4	4	*JG30-KF-CM45*	69	110	-37.2
5	*Gaiella*	185	170	8.8	5	*Solirubrobacter*	162	233	-30.5*
6	*Nocardioides*	150	142	5.6	6	*WD2101_soil_group*	122	163	-25.2
7	*MB-A2-108*	359	345	4.3	7	*0319-7L14*	93	122	-23.6
8	*MND1*	89	87	2.3*	8	*Vicinamibacteraceae*	165	214	-23.0
					9	*Pseudarthrobacter*	274	349	-21.4
					10	*Sphingomonas*	176	221	-20.4
					11	*Subgroup_7*	84	89	-5.6*
					12	*uncultured*	226	229	-3.0

Table S8. Relative abundance of fungal phyla in 2R and 2C（×10^4）

Upregulated					Downregulated				
	**fungal phyla**	**2R**	**2C**	**ΔC（%）**		**fungal phyla**	**2R**	**2C**	**ΔC（%）**
1	Kickxellomycota	61	5	1120.0	1	Rozellomycota	4	34	-88.2
2	Glomeromycota	251	62	304.8	2	Ascomycota	5863	7546	-22.3
3	Mortierellomycota	698	361	93.4	3	Zoopagomycota	62	78	-
4	unidentified	2376	1322	79.7					
5	Chytridiomycota	169	148	14.2					
6	Basidiomycota	578	522	10.7					

Table S9. Relative abundance of fungal phyla in 6R and 6C（$\times 10^4$）

Upregulated					Downregulated				
	**fungal phyla**	**6R**	**6C**	**ΔC （%）**		**fungal phyla**	**6R**	**6C**	**ΔC （%）**
1	Kickxellomycota	15	3	400.0	1	Zoopagomycota	1	11	-90.9
2	unidentified	1802	1044	72.6	2	Rozellomycota	6	14	-57.1
3	Glomeromycota	153	117	30.8	3	Mortierellomycota	343	644	-46.7
4	Basidiomycota	569	487	16.8	4	Chytridiomycota	156	266	-41.4
					5	Ascomycota	6956	7415	-6.2

Table S10. Relative abundance of fungal genera in 2R and 2C（×10^5）

Upregulated					Downregulated				
	**fungal genera**	**2R**	**2C**	**ΔC（%）**		**fungal genera**	**2R**	**2C**	**ΔC（%）**
1	*Rhizophagus*	267	3	8800.0	1	*Ramophialophor*	0	13	-100.0
2	*Pseudoacremonium*	117	5	2240.0	2	*Metarhizium*	27	4587	-99.4*
3	*Fusariella*	392	20	1860.0	3	*Mrakiella*	1	5	-80.0
4	*Mortierella*	6982	3606	93.6*	4	*Leptosphaeria*	173	718	-75.9
5	*Ascochyta*	1881	1019	84.6	5	*Cyphellophora*	3	8	-62.5
6	*Cephalotrichum*	18	10	80.0	6	*Lectera*	819	1770	-53.7
7	*Rhizophlyctis*	1146	830	38.1	7	*Fusarium*	416	829	-49.8
8	*unidentified*	41739	33002	26.5	8	*Vishniacozyma*	573	674	-15.0
9	*Paraphoma*	1897	1538	23.3	9	*Paramyrotheciu*	735	855	-14.0
10	*Epicoccum*	17177	13961	23.0					

Table S11. Relative abundance of fungal genera in 6R and 6C（×10^5）

	Upregulated					Downregulated			
	fungal genera	6R	6C	ΔC（%）		fungal genera	6R	6C	ΔC（%）
1	*Pseudoacremonium*	1035	1	103400.0	1	*Fusarium*	736	2202	-66.6
2	*Ramophialophora*	805	1	80400.0	2	*Metarhizium*	608	1273	-52.2
3	*Vishniacozyma*	980	17	5664.7	3	*Rhizophlyctis*	672	1319	-49.1
4	*Paramyrothecium*	11328	307	3589.9	4	*Mortierella*	3398	6407	-47.0*
5	*Mrakiella*	683	35	1851.4	5	*Paraphoma*	1248	2181	-42.8
6	*Rhizophagus*	829	55	1407.3	6	*unidentified*	50503	52615	-4.0
7	*Cyphellophora*	1666	644	158.7					
8	*Leptosphaeria*	1747	768	127.5					
9	*Fusariella*	2245	1008	122.7					
10	*Ascochyta*	3756	1723	118.0					
11	*Cephalotrichum*	785	502	56.4					
12	*Lectera*	690	449	53.7					
13	*Epicoccum*	5297	4974	6.5					

Table S12. Relative abundances of pathogenic microbial taxa in the rhizosphere soil of alfalfa（$\times 10^6$）

	**pathogenic microbial taxa**	**6R**	**6C**	**△C（%）**	**2R**	**2C**	**△C（%）**
1	*Pseudopeziza medicaginis*	390	0	-	0	0	
2	*Colletotrichum destructivum*	200	0	-	0	0	
3	*Paramyrothecium roridum*	113611	3061	3612.1	7426	8550	-13.1
4	*Fusarium oxysporum*	167	30	453.8	0	0	
5	*Fusarium proliferatum*	19855	24478	-18.9	913	4507	-79.7

Table S13. Relative abundances of beneficial microbial taxa in the rhizosphere soil of alfalfa（×**10^6**）

	**beneficial microbial taxa**	**6R**	**6C**	**△CK（%）**	**2R**	**2C**	**△CK（%）**
1	*Ensifer meliloti*	124	79	57.3	135	39	245.1*
2	*Pararhizobium giardinii*	53	96	-44.8	122	59	107.8*
3	*Bacillus sp.*	0	13	-100.0*	38	0	-
4	*Azospirillum sp.*	3	0	-	5	1	400.0
5	*Bacillus amyloliquefaciens*	0	1	-100	3	0	-

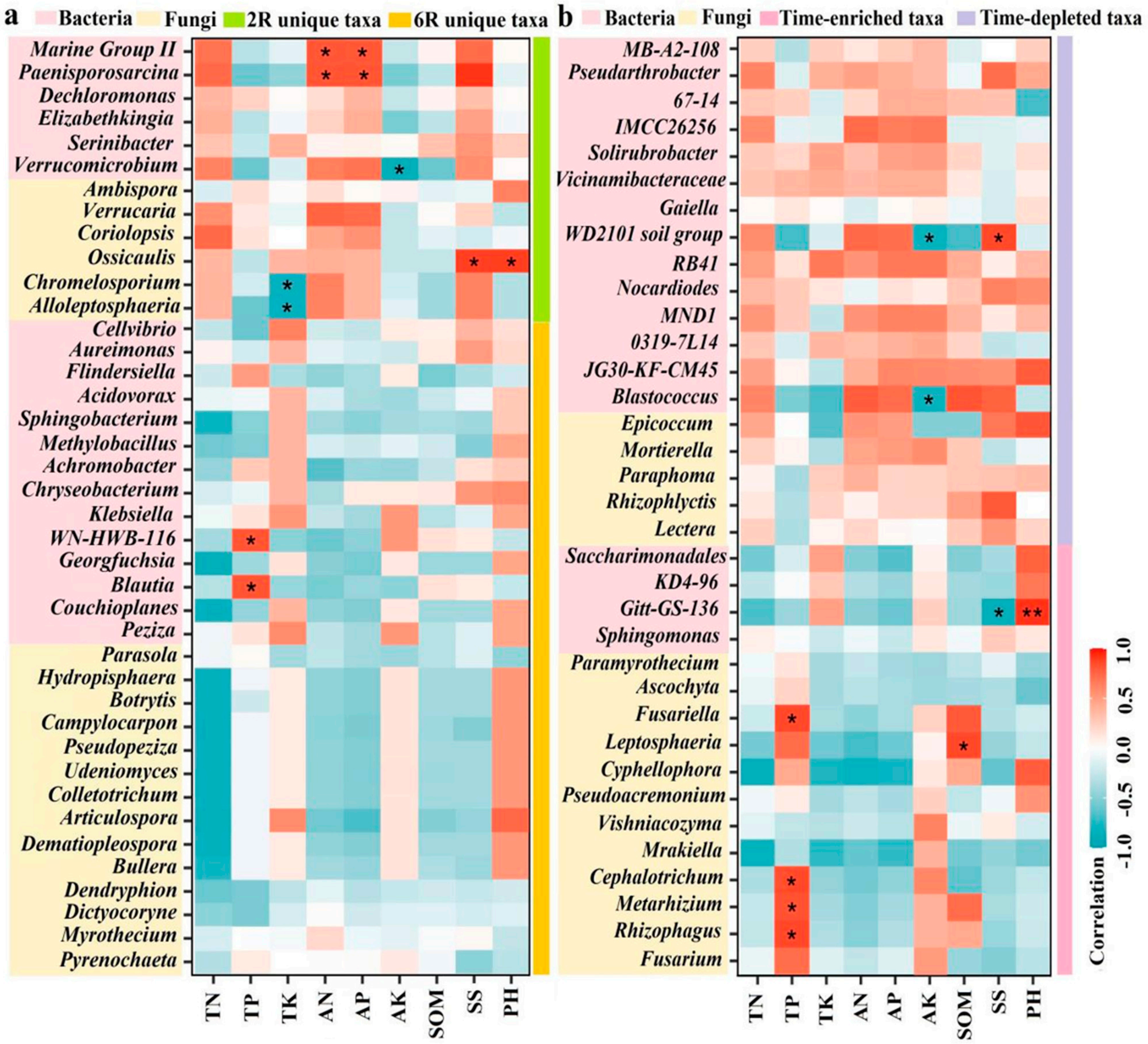

Figure S1 Correlation heatmaps between rhizosphere microbial taxa and soil properties in alfalfa stands. (a) Unique rhizosphere taxa; (b) Time-dependent taxa. 2R and 6R denote rhizosphere soils from the 2- and 6-year-old alfalfa stands, respectively. TN, total nitrogen; TP, total phosphorus; TK, total potassium; AN, alkali-hydrolyzable nitrogen; AP, available phosphorus; AK, available potassium; SOM, soil organic mater; SS, soil salt. * $P < 0.05$; ** $P < 0.01$.
